# TEG^®^ and ROTEM^®^ Traces: Clinical Applications of Viscoelastic Coagulation Monitoring in Neonatal Intensive Care Unit

**DOI:** 10.3390/diagnostics11091642

**Published:** 2021-09-07

**Authors:** Giulia Cannata, Elena Mariotti Zani, Alberto Argentiero, Caterina Caminiti, Serafina Perrone, Susanna Esposito

**Affiliations:** 1 Pediatric Clinic, Pietro Barilla Children’s Hospital, Department of Medicine and Surgery, University of Parma, via Gramsci 14, 43126 Parma, Italy; cannata.giulia@gmail.com (G.C.); e.mariottizani@gmail.com (E.M.Z.); alberto.argentiero@unipr.it (A.A.); 2 Research and Innovation Unit, University Hospital of Parma, via Gramsci 14, 43126 Parma, Italy; ccaminiti@ao.pr.it; 3 Neonatology Unit, Pietro Barilla Children’s Hospital, Department of Medicine and Surgery, University of Parma, via Gramsci 14, 43126 Parma, Italy; Serafina.perrone@unipr.it

**Keywords:** hemostasis, neonate, thromboelastography, thromboelastometry, viscoelastic testing

## Abstract

The concentration of the majority of hemostatic proteins differs considerably in early life, especially in neonates compared to adulthood. Knowledge of the concept of developmental hemostasis is an essential prerequisite for the proper interpretation of conventional coagulation tests (CCT) and is critical to ensure the optimal diagnosis and treatment of hemorrhagic and thrombotic diseases in neonatal age. Viscoelastic tests (VETs) provide a point-of-care, real-time, global, and dynamic assessment of the mechanical properties of the coagulation system with the examination of both cellular and plasma protein contributions to the initiation, formation, and lysis of clots. In this work, we provide a narrative review of the basic principles of VETs and summarize current evidence regarding the two most studied point-of-care VETs, thromboelastography (TEG^®^) and rotational thromboelastometry (ROTEM^®^), in the field of neonatal care. A literature analysis shows that viscoelastic hemostatic monitoring appears to be a useful additive technique to CCT, allowing targeted therapy to be delivered quickly. These tools may allow researchers to determine the neonatal coagulation profile and detect neonatal patients at risk for postoperative bleeding, coagulation abnormalities in neonatal sepsis, and other bleeding events in a timely manner, guiding transfusion therapies using the goal-oriented transfusion algorithm. However, diagnosis and treatment algorithms incorporating VETs for neonatal patients in a variety of clinical situations should be developed and applied to improve clinical outcomes. Further studies should be performed to make routinary diagnostic and therapeutic application possible for the neonatal population.

## 1. Background

Thromboelastography (TEG^®^) and rotational thromboelastometry (ROTEM^®^) are viscoelastic coagulation tests that quantify the process of clot formation and degradation. They both rapidly provide a graphical representation of the hemostatic process, including the interactions of platelets and fibrinolytic agents, leading to prompt and accurate therapeutic management [1,2,3,4,5]. The conventional coagulation test (CTT) performed in plasma including prothrombin time (PT), activated partial thromboplastin time (aPTT), thrombin time (TT), and fibrinogen partially reflects the interactions of all these elements, which contribute to clot formation and disruption; for this reason, the test does not seem to provide reliable information to guide transfusion or coagulation factor therapies [6,7,8,9,10].

A Neonatal Intensive Care Unit (NICU) patient often requires challenging management due to the underlying disease and needs swift hemostatic monitoring for the prevention of hemorrhagic and thrombotic complications. TEG^®^ and ROTEM^®^ constitute a simple method to evaluate the hemostatic profile in vivo with a very small sample of blood volume, representing a promising alternative to monitor coagulation in the neonatal population, although available TEG^®^ and ROTEM^®^ neonatal reference values are still very limited [11,12,13].

In this narrative review of the literature, we analyze the basic principles of viscoelastic hemostatic assays (VHAs) and current evidence-based clinical applications of TEG^®^ and ROTEM^®^ in the field of NICU. This review was carried out by the Department of Pediatrics in Parma. Systematic searches were performed in PubMed, Embase, Cochrane Library, Scopus, Google Scholar, and ClinicalTrials.gov up to 10 May 2021. Language was restricted to English. Search terms included viscoelastic testing (VET), thromboelastography (TEG^®^), and rotational thromboelastometry (ROTEM^®^) in combination with neonate, newborn, infant, and preterm infant. Case reports, case series, original research studies, review articles, letters to the editor, randomized controlled trials (RCTs), non-RCTs, and cohort studies (prospective or retrospective) were included.

## 2. Methods

### 2.1. Thromboelastography (TEG^®^)

**Figure 1:**
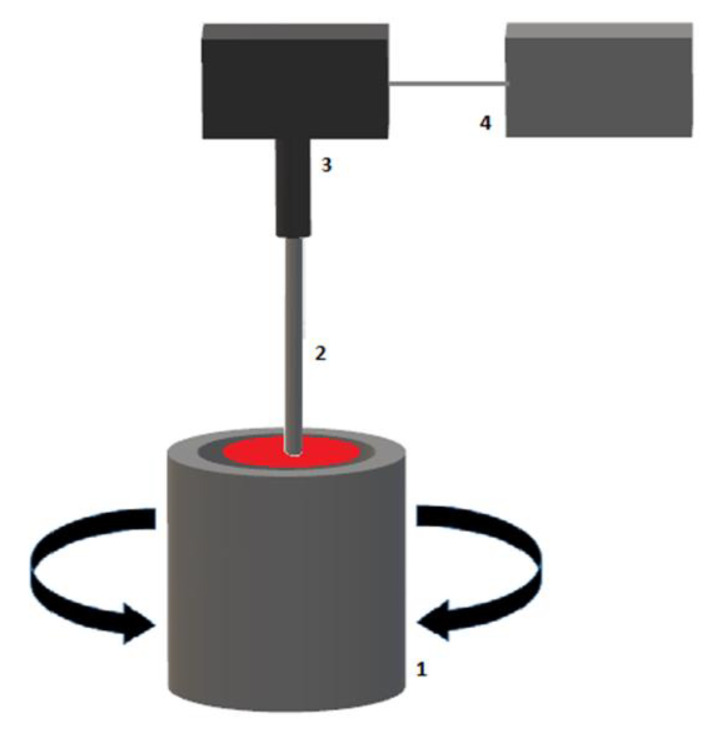
Schematic illustration of a TEG^®^ device. (1) Blood sample in rotating cup, (2) pin and torsion wire, (3) electromechanical transducer, (4) data processing unit.

TEG^®^ is performed with a 360 μL whole blood sample (TEG^®^ 5000 Thrombelastograph^®^ Hemostasis Analyzer system). The blood sample is placed in a heated (37 °C) cylindrical cup that rotates with an angle of 4°45′ and a cycle duration of 10 s, simulating venous flow. A pin is immersed into the blood sample and suspended by a torsion wire connected to a mechanical–electrical transducer and subsequent data-processing unit (Figure 1) [14,15,16,17].

At the onset of each measurement, whole blood behaves as a non-Newtonian fluid, and the viscous shear between the rotating cup and stationary pin results in no torque and absent deflection in the viscoelastic trace.

**Figure 2:**
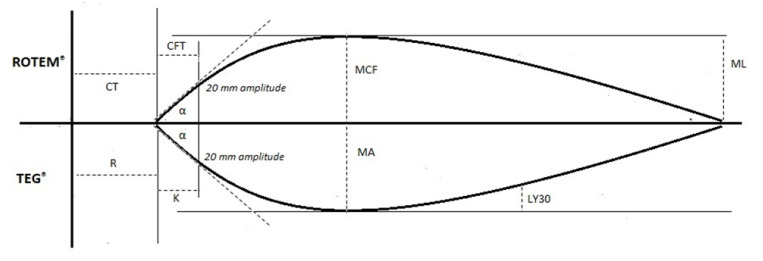
Illustration of thromboelastography (TEG^®^) tracing and the accompanying parameters. Abbreviations: R: reaction time; K: kinetics time; α: alpha angle; MA: maximum amplitude; LY30: lysis at 30 min.

Once a clot starts to form and grows in strength, the whole blood within the rotating cup begins its transition from a viscous to elastic state; the resultant rotational force exerted on the immersed pin causes rotation on its axis, which is tracked and plotted as a deflection from the baseline to maximum amplitude (maximum cloth strength). Once clot lysis begins, the deflection returns back toward the baseline [18,19]. Results are graphically represented, with the amplitude of motion detected via torsion wire and an electromagnetic transducer plotted against time. A typical TEG^®^ trace is shown (Figure 2).

TEG^®^ parameters depict a coagulation profile representing the cell-based model of hemostasis, including the stages of initiation, amplification, propagation, and termination through fibrinolysis (Table 1) [8]. Different available reagent kits provide additional information on hemostatic pathways (Table 2) [14,15,16,17,20,21].

### 2.2. Thromboelastometry (ROTEM^®^)

**Figure 3:**
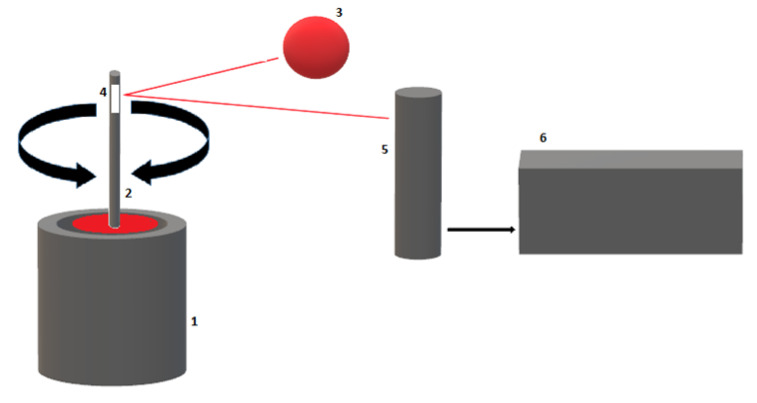
Schematic illustration of a ROTEM^®^ device. (1) Cuvette with blood sample, (2) oscillating axis, (3) LED light source, (4) mirror, (5) detector, (6) data processing unit.

ROTEM^®^ is performed with a 300 μL citrated whole blood sample (ROTEM^®^ delta Thromboelastometry systems). The blood sample is placed in a fixed and heated (37 °C) cup; contrary to the TEG^®^, the immersed pin makes a 4°75′ rotation. As the clot builds up, the rotation of the pin is restricted in proportion to the clot strength; pin movement is detected by an optical sensor (Figure 3).

**Figure 4:**
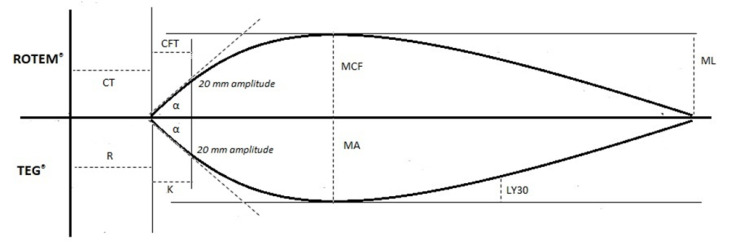
Illustration of thromboelastometry (ROTEM^®^) tracing and the accompanying parameters. Abbreviations: CT: clotting time; CFT: clot formation time; α: alpha angle; MCF: maximum clot firmness; ML: maximum lysis.

The graphical tracing of cloth strength is plotted as movement amplitude relative to the time elapsed from the initiation of the coagulation cascade to fibrinolysis (Figure 4) [16,17,22].

VET results are graphically represented, with the amplitude of movement originating from the rotating pin detected via an optical detector plotted against time. A typical ROTEM^®^ trace is illustrated in Figure 4. ROTEM^®^ parameters provide global information about the kinetics of clot formation and dissolution, including the stages of initiation, amplification, propagation, and termination through fibrinolysis (Table 1) [6,23].

A wide range of assays is available for the functional investigation of the hemostatic and fibrinolytic systems (Table 3) [16,17,20,21,22].

## 3. Developmental Hemostasis: The Neonatal Coagulation System

The hemostatic system is a dynamic, evolving system that changes and matures over time from fetal to adult life. Physiological, age-dependent changes of the coagulation system are referred to as developmental hemostasis [24,25,26,27,28].

Hemostasis is an active, interconnected network of processes balancing the simultaneous and opposing forces of coagulation and anti-coagulation. The holistic cell-based coagulation model involves both cellular and plasma protein components interacting together in the four overlapping phases of initiation, amplification, propagation and termination. Multiple enzymes, cofactors, adhesion molecules, receptors, platelets, and cellular elements including red blood cells, white cells, the endothelium, and extravascular fibroblasts play a key role in this process [6,29].

Maternal coagulation factors are unable to cross the placental barrier because of their size [30,31,32]; fetal synthesis of coagulation proteins begins at 11 weeks of gestation. Physiological concentrations of coagulation proteins gradually increase with time; levels are lower in premature infants compared to full-term babies and healthy children [25,26,27,33,34,35,36,37].

Adult plasma levels of most coagulation proteins are reached between a few months of age and 16 years old. Neonatal plasma concentrations of vitamin K-dependent coagulation factors (II, VII, IX, X) and contact factors (XII, XI, high-molecular-weight kininogen and prekallikrein) are about 50% of adult values [24,25,37]. However, in contrast, the major inhibitors of the coagulation system antithrombin (AT), protein C (PC), and protein S (PS) show low levels at birth. Thrombin generation and fibrinolytic capacity are reduced (Table 4) [26,38,39,40,41].

The neonatal platelet count and mean platelet volume are usually normal or high, reaching adult values within 1 year. However, platelet hyporeactivity has been reported in newborns; potential reasons reported include impaired signal transduction, the deficiency of thromboxane synthesis, and a decreased density of platelet receptors [42,43,44,45].

Overall, a shorter primary hemostasis bleeding time, reflecting an interaction between platelets and damaged endothelial cells to form a platelet plug, was found in healthy neonates compared with adults, which was normalized before the first month of life [46,47,48,49]. Reasons reported are an increased concentration and activity of von Willebrand factor (vWf; i.e., a high-molecular-weight adhesive glycoprotein that plays an essential role in primary hemostasis) potentiating vWF-mediated platelet adhesion and high hematocrit with the presence of large nucleated red cells [24,37,50,51].

## 4. Viscoelastic Testing Results from Healthy Neonates

### 4.1. TEG^®^/ROTEM^®^ Results from Cord Blood Samples

Published data about VET in a healthy neonatal population are limited and mainly based on cord blood samples.

Recalcified citrate cord blood TEG^®^ reference intervals were established by Edwards et al. The hemostatic profile of healthy neonates displayed by TEG^®^ showed a significantly shorter reaction time (R-time) (*p*-value < 0.001) as opposed to children and adults and higher values for alpha angle (α angle) than for children (*p*-value < 0.001), with a moderately higher maximum amplitude (MA) as opposed to children (*p*-value < 0.05). The coagulation index (CI) (CI= −0.2454R+ 0.0184K + 0.1655MA − 0.0241a − 5.0220) and the G parameter (G) (shear elastic modulus strength or clot strength) were higher compared to children (*p*-value < 0.001), while G values were significantly lower compared to adult controls (*p*-value < 0.001) [52].

NATEM ROTEM^®^ analysis with an increasing concentration of tissue plasminogen activator (tPA) performed on neonatal cord blood samples by Sidlik et al. similarly described the accelerated initiation and propagation of coagulation compared to adults along with increased clot firmness and enhanced fibrinolysis in comparison to children. Neonatal CT and CFT were shorter compared to adults (*p*-value ≤ 0.002), while alpha angle (α angle) values were higher than those for adults (*p*-value = 0.002). Increasing concentrations of tissue plasminogen activator (tPA) correlated with a significant decrease in maximum clot firmness (MCF) (*p*-value = 0.0001) [53].

ROTEM^®^ was used for the first time to assess clot formation in preterm infants by Strauss et al. [54]. Modified EXTEM ROTEM^®^ analysis performed on full-term neonatal cord blood samples showed accelerated clot formation kinetics characterized by significantly shorter CT and CFT compared to adult controls (*p*-value = 0.001). Modified EXTEM ROTEM^®^ analysis performed on preterm neonatal cord blood samples showed shorter CT (*p*-value = 0.001) and CFT (*p*-value = 0.002) compared to adults. Maximum clot firmness (MCF) values were lower than those for full term neonates (*p*-value = 0.004) and adults (*p*-value = 0.001). They also showed a significant correlation between CT and maximum clot firmness (MCF) values and gestational age (GA) in preterm newborns (Pearson correlation r = 0.132, *p*-value = 0.045 and r = 0.259, *p*-value < 0.001, respectively) [54].

Cvirn et al., using thromboelastometry with an extrinsic activator assay, recorded no difference in coagulation times between cord blood samples and adult controls. Maximum clot firmness (MCF) values were significantly lower in neonates compared to adults, which was probably due to an impaired polymerization of neonatal fibrin [55].

Wiegele et al. have recently published reference ranges in preterm (30 + 0 to 36 + 6 weeks/days) and term neonates (37 + 0 to 39 + 6 weeks/days) delivered by cesarian section for all types of commercially available thrombelastometry tests [56].

Reference values for cord blood TEG^®^ in full-term healthy newborns were established by Mirabella et al. [57].

Finally, no statistical differences (*p* < 0.01) were observed by Schott et al. in TEG measurements (reaction time, kinetics time, α angle, maximum amplitude) between healthy neonates following vaginal and cesarean section deliveries [58].

Table 5 summarizes the main TEG®/ROTEM® results obtained in the neonatal population.

### 4.2. TEG^®^/ROTEM^®^ Results from Newborns’ Blood Samples

Scarce data exist about the hemostatic profiles assessed for peripheral blood samples with VETs in healthy neonates. Caution must be taken when interpreting experimental data due to the lack of homogeneity in commercially available viscoelastic hemostatic assays and the reagents used, the small size samples, and the variability of age ranges included in studies.

Motta et al. established reference values for citrated-native whole blood TEG^®^ assay in 65 healthy preterm neonates (gestational age at birth < 37 weeks). Blood samples were collected within 36 h after birth; exclusion criteria were the following: death within 7 days of life, early-onset sepsis confirmed by a positive blood culture, suspected sepsis with raised C-reactive protein defined as values >10 mg/L, presence of bleeding (intracranial, gastrointestinal, cutaneous, and pulmonary hemorrhage), platelet count < 100 × 109 /L. Reference intervals were established in early-preterm neonates (gestational age at birth < 32 weeks; 32 neonates included in the group) and moderate/late-preterm neonates (gestational age at birth from 32 to 37 weeks; 33 neonates included in the group). Similar TEG^®^ parameters values were found comparing early-preterm neonates with moderate/late-preterm neonates: reaction time (R-time) (*p*-value = 0.840), kinetics time (K-time) (*p*-value = 0.681), alpha angle (α angle) (*p*-value = 0.830), and maximum amplitude (MA) (*p*-value = 0.984) parameters did not statistically differ among the two groups. The hemostatic profile of healthy moderate/late-preterm neonates displayed by TEG^®^ showed a lower coagulation index (CI) (*p*-value = 0.847) and higher values of the G parameter (G) (shear elastic modulus strength, or clot strength) (*p*-value = 0.984). TEG^®^ analysis performed on early-preterm neonatal blood samples showed statistically significant higher values of lysis at 30 min (LY30) compared to moderate/late-preterm neonates (*p*-value = 0.013) [59].

Native-blood TEG^®^ analysis was used to assess clot formation in 237 healthy pediatric patients of less than 2 years of age undergoing elective non-cardiac surgery by Miller et al.; a total of 37 term infants were enrolled. Exclusion criteria were the use of drugs known to interfere with blood coagulation, which is a previously diagnosed congenital heart disease and ongoing acute systemic disease. Blood samples were drawn after the induction of general anesthesia. Reaction time (R-time), kinetics time (K-time), alpha angle (α angle), and amplitude of TEG^®^ tracing 60 min after maximum amplitude (A-60) parameters were measured from the TEG^®^ tracing. Neonatal TEG^®^ values did not statistically differ from those of older patients (aged from 1 to 24 months). When compared to adult control subjects, native-blood TEG^®^ analysis performed on pediatric patients of less than 12 months of age described an accelerated initiation (R-time) and propagation of coagulation (K-time, α angle) along with increased clot strength (MA) [60].

Sewell et al. established reference ranges for citrated-modified and heparinase-modified TEG^®^ assay after kaolin activation in 30 term newborns admitted to the NICU and diagnosed with known or suspected congenital surgical anomalies, neurosurgical diseases, urological abnormities, and non-critical congenital heart diseases. Exclusion criteria were a personal or family history of bleeding disorders and known or suspected major chromosomal anomaly [61].

Sokou et al. established reference values for peripheral arterial whole blood EXTEM ROTEM^®^ assay in 198 full-term neonates (gestational age at birth ≥ 37 weeks) and 84 preterm newborns (gestational age at birth < 37 weeks). Exclusion criteria were a personal history of perinatal blood loss, birth asphyxia or perinatal stress, a known or suspected major chromosomal anomaly, a family history of bleeding disorders, and ongoing septicemia. Comparable EXTEM ROTEM^®^ parameters values were found comparing full-term neonates with preterm newborns: CT (*p*-value = 0.19), CFT (*p*-value = 0.06), alpha angle (α angle) (*p*-value = 0.45), amplitude 10 min after CT (A10) (*p*-value = 0.90), amplitude 20 min after CT (A20) (*p*-value = 0.15), and MCF (*p*-value = 0.17) parameters did not statistically differ among the two groups. An enhanced fibrinolytic activity in preterm neonates was observed: lysis at 60 min (LY60) values were significantly lower than those for full-term neonates (*p*-value = 0.006). The authors also demonstrated a significant correlation between lysis at 60 min (LY60) and GA and birth weight in preterm newborns (Spearman correlation coefficient ρ =−0.3763, *p*-value = 0.002 and Spearman correlation coefficient ρ =−0.2988, *p*-value = 0.016, respectively) [62].

EXTEM ROTEM^®^ analysis was used for the first time by Sokou et al. to assess clot formation in small for gestational age (SGA) neonates in comparison to appropriate for gestational age weight (AGA) neonates. SGA newborns are defined as having a birth weight of less than the 10th percentile for gestational age. A total of 93 neonates were enrolled in the study: the SGA group included 23 full-term newborns and 22 preterm newborns, while the AGA group included 23 full-term newborns and 25 preterm newborns. Exclusion criteria included a personal history of significant bleeding events, transfusion of blood, platelets, fresh frozen plasma or cryoprecipitate, a family history of bleeding disorders, birth asphyxia, moderate or severe acidemia, ongoing septicemia, and a known or suspected major chromosomal anomaly. Comparable EXTEM ROTEM^®^ parameters values were found comparing small for gestational age (SGA) neonates with appropriate for gestational age weight (AGA) neonates: CT (*p*-value = 0.36), CFT (*p*-value = 0.29), amplitude 5 min after CT (A5) (*p*-value = 0.45), amplitude 10 min after CT (A10) (*p*-value = 0.43), alpha angle (α angle) (*p*-value = 0.18), MCF (*p*-value = 0.54), and lysis at 60 min (LY60) (*p*-value = 0.21) values did not statistically differ among the two groups. Moreover, no significant statistical differences were noticed in any of the EXTEM ROTEM^®^ parameters with regard to delivery time when comparing SGA full-term neonates to AGA full-term neonates and SGA preterm neonates to AGA preterm neonates [63].

Ravn et al. performed whole blood EXTEM, INTEM, and FIBTEM ROTEM^®^ measurements on 149 pediatric patients of less than 72 months of age; a total of 23 healthy neonates and seven neonates scheduled for congenital cardiac surgery were enrolled. Age-specific reference values for EXTEM, INTEM, and FIBTEM ROTEM^®^ assays were developed, including those of the neonatal group [64].

Theodoraki et al. established reference ranges for EXTEM, INTEM, and FIBTEM ROTEM^®^ assays performing measurements on 215 healthy term neonates with a median gestational age of 39 weeks. Reference ranges were obtained for clotting time (CT), clot formation time (CFT), α-angle, clot firmness at 10 min (A10), maximum clot firmness (MCF), and lysis index at 60 min (LI60, %) [65].

### 4.3. Viscoelastic Testing in Sick Neonatal Population

TEG^®^ and ROTEM^®^ have been extensively studied and applied in adult patients, especially in major bleeding related to severe trauma and surgical fields, such as liver transplantation as well as cardiac and obstetric procedures. Their use has been largely considered in the management of trauma-induced coagulopathy because of their great precision to identify the lacking element involved in the hemostasis reaction [66,67,68,69,70,71,72,73,74,75,76,77,78].

Neonatal data are very limited because of the scarcity of viscoelastic reference values, the extreme variability of the NICU patients, and the differences in coagulation status, which is influenced by gestational age, weight, and maturation of hepatic functions [24,25,26,27,28,33,34,35,36,37]. TEG^®^ and ROTEM^®^ clinical applications in children have been almost entirely anesthesiology-driven, especially in newborn candidates for surgery, including cardiac procedures and ExtraCorporeal Membrane Oxygenation (ECMO) [79,80]. With regard to neonatology, the main application areas include neonatal sepsis, intraventricular hemorrhage (IVH), hypoxic–ischemic encephalopathy (HIE), and newborn candidates for therapeutic hypothermia [11,12].

Thromboelastometry is an efficient tool to assess the hemostatic efficacy of fibrinogen infusions in patients with quantitative fibrinogen disorders undergoing major surgical procedures [81]. Most patients with hypofibrinogenemia are asymptomatic, while others show signs of severe bleeding events (e.g., umbilical cord bleeding, intracranial hemorrhage, splenic rupture, or bleeding episodes induced by trauma or surgery [82,83,84]). A recent study conducted by Sirmuda et al. observed a reduced level of fibrinogen in a 6-year-old boy with delayed bleeding after 6 h of cleft lip surgery and a 34-year-old woman diagnosed with mild hypofibrinogenemia detected in the first trimester of her second pregnancy with further complications due to retroplacental hematoma. Laboratory analysis confirmed mild hyperfibrinolysis, and in both cases, a new nonsense mutation in the FGB gene was detected, leading to mild hypofibrinogenemia [23].

Thromboelastometric evaluation of coagulation in children undergoing cardiac surgery is associated with a reduction in postoperative blood loss and transfusion requirements and shorter hospitalization [85,86]. Neonates undergoing CPB are at an increased risk of bleeding events due to their immature coagulation system. The prospective observational study conducted by Scott et al. on 44 NICU patients undergoing cardiopulmonary bypass (CBP) concluded that ROTEM^®^ can rapidly detect thrombocytopenia and hypofibrinogenemia and consequently implement measures that may improve peri-CPB hemostasis and minimize transfusion-related complications such as venous thromboembolism and transfusion-related acute lung injury. EXTEM A10 can predict thrombocytopenia and FIBTEM A10 can predict hypofibrinogenemia in neonates undergoing CPB. The diminished clot strength in neonatal age is probably the result of the impaired polymerization properties of the neonatal fibrin and the increased fibrinolytic activity due to the increased tissue-plasminogen activator (tPA) levels and the reduced levels of the fibrinolysis inhibitors plasminogen activator inhibitor (PAI) and α2-antiplasmin [87].

Moreover, in surgical settings, the administration of fresh frozen plasma (FFP) is a realistic alternative to normalize hemostasis [88], and fibrinogen replacement could be guided by MCF values using rotational thromboelastometry [89].

TEG^®^ and ROTEM^®^ may detect coagulation status abnormalities in newborns with sepsis. The study conducted by Sakou et al. on 91 septic neonates concluded that hypercoagulation, but especially hypocoagulation, represents an early finding of sepsis and seems to be the main coagulopathy associated with neonatal sepsis [90]. The explanations of this phenomenon lie in the evidence that vitamin K-dependent factors are reduced to 50% of the normal value reached in adults. Moreover, neonatal platelets have a decreased granule secretion and expression of fibrinogen-binding sites [47]. All these factors play a role in the promotion of a pro-hemorrhagic status, especially in sick neonates. The degree of the disruption of the hemostatic balance might be correlated with the gravity of sepsis [90]. Nevertheless, limited data are available about the role of viscoelastic tests as an indicator of early neonatal sepsis, and further studies should be performed.

Perinatal hypoxia and asphyxia represent a serious issue that neonatologists are confronted with in clinical practice; one of the dysfunctions caused by the interruption of fetal blood flow and impairment of gas exchange is the derangement of the hemostatic balance, predisposing to hemorrhage and/or thrombosis [91]. A recent study conducted by Konstantinidi et al. evaluated the hemostatic profiles of 164 neonates with perinatal hypoxia and compared them to healthy controls using viscoelastic whole blood tests (EXTEM ROTEM^®^ analysis). The authors demonstrated a hypocoagulable profile in neonates with perinatal asphyxia or fetal distress compared to healthy neonates. EXTEM ROTEM^®^ analysis performed on hypoxic neonates showed prolonged CT and CFT compared to healthy neonates (*p*-value < 0.001); alpha angle (α angle) and maximum clot firmness (MCF) values were lower than those of healthy newborns (*p*-value < 0.001). EXTEM ROTEM^®^ analysis performed on asphyxiated neonates showed a significantly prolonged CT (*p*-value = 0.005) and CFT (*p*-value = 0.003) and reduced alpha angle (α angle) (*p*-value = 0.028) compared with newborns with fetal distress. These findings indicate that this test potentially may be used to explore coagulation in hypoxic neonates further [92]. In addition, a hypoxic event and the subsequent reperfusion injury can result in a thromboxane and thrombopoietin release, affecting platelet function [91].

A study conducted by Pakvasa et al. on 98 neonates with hypoxic–ischemic encephalopathy (HIE) measured PT, platelet count, and fibrinogen concentration. Initial hemostatic dysfunction was defined by the authors as one or more of the following: PT ≥ 18 s, platelet count <100 × 103 /μL, or fibrinogen concentration < 150 mg/dL. The prevalence of initial hemostatic dysfunction was 69% (95% CI 59% to 78%); 27 neonates (28%; 95% CI 19% to 38%) had abnormal bleeding events, 56 (57%) received at least one blood product transfusion, and three neonates died from bleeding complications [93]. These findings suggest that hemostatic dysfunction is prevalent and associated with an increased risk of bleeding and high transfusion burden, and the assessment of initial hemostatic profiles should be routinely performed in all newborns with moderate-to-severe HIE.

In conclusion, in the light of all the above, viscoelastic tests may be useful tools to determine the neonatal coagulation profile and detect neonatal patients at risk of developing postoperative bleeding, coagulation abnormalities in neonatal sepsis and other bleeding events in a timely manner, guiding transfusion therapies using the goal-oriented transfusion algorithm.

Table 6 shows the main TEG®/ROTEM® results obtained in the sick neonatal population.

## 5. Conclusions

Hemostasis is a dynamic process that changes and matures over time from fetal to adult life. The concept of developmental hemostasis is critical to ensure the optimal diagnosis and treatment of hemorrhagic and thrombotic diseases in pediatric patients, especially in neonates [24,25,26,27,28]. Healthy neonates are born with an apparent combined deficiency in plasma coagulation factors, natural inhibitors of hemostasis, and components of the fibrinolytic system directly dependent on gestational age, birth weight, and maturation of hepatic function. Neonatal plasma concentrations of vitamin K-dependent coagulation factors (II, VII, IX, X) and contact factors (XII, XI, high-molecular-weight kininogen and prekallikrein) are about 50% of adult values and reach adult plasma levels between a few months of age and 16 years old [24,25,37]. Nevertheless, the hemostatic system is functionally balanced with no tendency toward coagulopathy or prothrombosis [26,38,39,40,41,46,47,48,49,50,51]. This delicate balance is overturned in sick neonates, with CCT appearing not to be completely suitable to ensure the optimal diagnosis and treatment of the main neonatal hemorrhagic and thrombotic diseases.

The approach to coagulation disorders in newborns in current clinical practice includes standard coagulation tests, such as PT, aPTT, and TT as well as fibrinogen and platelet count. TEG^®^ and ROTEM^®^ both provide real-time whole-blood measurements of hemostatic process kinetics, providing a global rapid assessment of clotting, platelet function, and fibrinolysis. Compared to CCT, viscoelastic tests more closely reflect the in vivo hemostatic conditions. The availability of different reagents and simultaneous sample investigation allow a more accurate and specific evaluation of the hemostatic cascade and permit researchers to investigate clot initiation, propagation, stabilization, and dissolution processes separately.

Viscoelastic hemostatic monitoring has been rapidly expanding and appears to be a useful additive technique to CCT, allowing targeted therapy to be delivered quickly. These may be useful tools to determine the neonatal coagulation profile and detect neonatal patients at risk of developing postoperative bleeding, coagulation abnormalities in neonatal sepsis, and other bleeding events in a timely manner, guiding transfusion therapies using the goal-oriented transfusion algorithm. However, TEG^®^ and ROTEM^®^ reference data are scarce in the neonatal population. Diagnosis and treatment algorithms incorporating VETs for neonatal patients in a variety of clinical situations should be developed and applied to improve clinical outcome. However, most available data come from small-sample-size studies, differing in study designs both in terms of patient inclusion criteria and the whole blood viscoelastic assays performed. Further studies on homogeneous and larger study populations should be performed to make routinary diagnostic and therapeutic application possible for the neonatal population.

## Figures and Tables

**Table 1:** Comparison of TEG^®^ and ROTEM^®^ parameters and relative physiological significance.

TEG^®^ Test Variable	Definition	ROTEM^®^ Test Variable	Definition	Physiological Significance
Reaction time (R-time)	Time until clot amplitude of 2 mm is reached	Clotting time (CT)	Time until clot amplitude of 2 mm is reached	Initiation phase of enzymatic clotting factor activation. It is a measure of time taken to initiate coagulation
Kinetics time (K-time)	Time until clot amplitude of 20 mm is reached (from 2 mm amplitude)	Clot formation time (CFT)	Time until clot amplitude of 20 mm is reached (from 2 mm amplitude)	The amount of time it takes to reach a certain clot strength (amplitude of 20 mm)
Alpha angle (α)	Angle between central horizontal line and a tangent to the curve through the 20 mm amplitude point	Alpha angle (α)	Angle between central horizontal line and a tangent to the curve through the 20 mm amplitude point	Rate of clot formation and strengthening (kinetic measurement of fibrin–platelet interaction)
		Amplitude 10 min after CT (A10)	Amplitude at 10 min after clotting time	Measure of clot strength (fibrin–platelet interaction)
		Amplitude 30 min after CT (A30)	Amplitude at 30 min after clotting time	Measure of clot strength (fibrin–platelet interaction)
Maximum amplitude (MA)	Peak amplitude of clot	Maximum clot firmness (MCF)	Peak amplitude of clot	Measure of clot strength (fibrin–platelet interaction
Lysis at 30 min (LY30)Lysis at 60 min (LY60)	Percentage decrease in clot strength at 30 min after maximum amplitude (MA) Percentage decrease in clot strength at 60 min after maximum amplitude (MA)	Maximum lysis (ML)	Maximum percentage reduction in maximum clot firmness (MCF)	Measure of clot stability. Fibrinolytic-induced dissolution of the fibrin–platelet bond

**Table 2:** Commercially available TEG^®^ assays.

TEG^®^ Test	Activator	Rationale
Native TEG	None	Assessment of native blood coagulation
Kaolin TEG	Kaolin	Test of intrinsic pathway (Kaolin-mediated activation of factor XII). Faster results than the native test
Kaolin TEG	Kaolin and tissue factor	Test of both intrinsic and extrinsic pathways (Tissue factor-dependent activation of factor VII). Rapid assessment of blood coagulation
Kaolin TEG with heparinase	Kaolin and heparinase	Heparinase inactivates heparin. Assessment of heparin reversal on blood coagulation
TEG Functional Fibrinogen	Tissue factor and abciximab	Abciximab acts as a platelet GpIIb/IIIa inhibitor. Test of extrinsic pathway allowing for the quantification of fibrinogen contribution to clot strength after platelet inhibition

**Table 3:** Commercially available ROTEM^®^ assays.

ROTEM^®^ Test	Activator	Rationale
NATEM	None	Assessment of native blood coagulation
INTEM	Phospholipid and ellagic acid	Ellagic acid acts as intrinsic pathway activator. Test of intrinsic pathway, more sensitive to intrinsic pathway factor deficiencies
EXTEM	Tissue factor	Test of extrinsic pathway (tissue factor-dependent activation of extrinsic pathway). More sensitive to extrinsic pathway factor deficiencies. Fastest clot analysis
HEPTEM	Phospholipid, ellagic acid, and heparinase	Heparinase inactivates heparin. Assessment of heparin reversal on blood coagulation
APTEM	Tissue factor and aprotinin	Aprotinin inhibits fibrinolysis. Test of fibrinolysis
FIBTEM	Tissue factor and cytochalasin D	Cytochalasin D acts as a platelet inhibitor. Quantification of fibrinogen contribution to clot strength after platelet inhibition

**Table 4:** Coagulation parameters in neonatal and childhood vs. adult periods.

Parameter	Neonatal Period (Mean Value)	Normalization
Platelets	Normal or increased	1 year (after transient increases)
von Willebrand factor (vWF)	Increased (153%)	3 months
FII	Decreased (40–66%)	1 year
FVII	Decreased (40–66%)	1 year (up to 16 years)
FIX	Decreased (40–66%)	1 year
FX	Decreased (40–66%)	1 year
FXI	Decreased (37–54%)	1 year
FXII	Decreased (37–54%)	1 year
FV	Normal or decreased (70%)	1 year (up to 16 years)
FVIII	Normal or increased (100%)	1 month
Prekallikrein (PK)	Decreased (37–54%)	1 year
High-molecular-weight kininogen (HMWK)	Decreased (37–54%)	1 year
Fibrinogen	Decreased or normal	1 year
Antithrombin (AT)	Decreased (63%)	3 months
Protein C (PC)	Decreased (35%)	16 years
Protein S (PS)	Decreased (36%)	3 months
Plasminogen	Decreased (36%)	6 months
Alpha 2 antiplasmin	Normal or decreased (85%)	6 months
Tissue plasminogen activator (tPA)	Increased	1 week
D-dimer	Increased	16 years

AT: antithrombin; HMWK: high-molecular-weight kininogen; PK: prekallikrein; PC: protein C; PS: protein S; tPA: tissue plasminogen activator; vWF: von Willebrand factor.

**Table 5:** Main TEG®/ROTEM® results obtained in the healthy neonatal population: study characteristics.

Author	Included Neonates (n)	Control Group	Type of blood Sample	Analyzing Method	Findings
Wiegele et al. [56]	142 neonates. 55 preterm infants, 87 full-term infants		Cord blood	ROTEM	Significantly faster clot initiation and formation as well as higher clot strength in the term group
Schott et al. [58]	100 full-term neonates.50 delivered vaginally; 50 delivered by cesarean section		Cord blood	TEG	No differences between vaginal and cesarean delivery neonates in TEG measurements
Mirabella et al. [57]	85 full-term neonates	40 adults	Cord blood	TEG	No between neonatal and adult TEG parameters No differences between neonatal and adult TEG value ranges
Sidlik et al. [53]	101 neonates	Adults	Cord blood	ROTEM	Lower CT and CFT values and higher alpha angle in neonates (faster clot formation compared to adults). Accelerated fibrinolysis in the newborns compared to adults (shorter LI30)
Strauss et al. [54]	231 (84 full-term and 47 preterm infants)	Institution’s reference ranges for adults and children	Cord blood	ROTEM	CT and CFT significantly shorter among preterm and term infants compared to adults (faster clot formation) Decreased MCF in preterm compared to term neonates and adults. Correlation between GA and CT and MCF
Edwards et al. [52]	59 neonates (>34 weeks)	Institution’s reference ranges for adults and children	Cord blood	TEG	Accelerated initiation of coagulation and increased clot firmness and enhanced fibrinolytic activity compared to children (shorter R, higher angle, MA, CI, and G values) Accelerated initiation and propagation of coagulation compared to adults (shorter R, lower G values)
Cvirn et al. [55]	20 full-term neonates	20 adults	Cord blood	ROTEM	Lower MCF and α angle and longer CFT (FIBTEM) in neonates compared to adults

**Table 6:** Main TEG®/ROTEM® results obtained in the sick neonatal population: study characteristics.

Author	Included Neonates (n)	Type of Blood Sample	Analyzing Method	Findings
Theodoraki et al. [65]	215 full-term neonates	Whole blood	ROTEM	Positive correlation between LY30, LY45, and LY60 variables of EXTEM and INTEM assays and gestational age. ROTEM neonatal variables not influenced by maternal problems during pregnancy and delivery mode. Reduced A5 in INTEM and prolonged CT and CFT in INTEM and EXTEM assays were observed in neonates with higher hematocrit levels
Raffaeli et al. [94]	60 neonates	Whole blood and cord blood	TEG	Placental blood leads to a procoagulant imbalance when testing is performed with TEG
Sokou et al. [63]	<37 weeks SGA: 22 <37 weeks AGA:25 ≥37 weeks SGA: 23 ≥37 weeks AGA: 23	Whole blood	ROTEM	No statistically significant differences were noticed regarding all EXTEM parameters between AGA and SGA neonates
Raffaeli et al. [95]	283 neonates VLBW: 201, ≥37 weeks: 72	Whole blood	TEG	Healthy VLBWIs showed TEG profiles suggesting a relatively balanced hemostatic system, with slight hypocoagulability initially (compared with term neonates), gradually evolving to a somewhat more procoagulant phenotype over the first month
Liu et al. [96]	371 full-term neonates	Whole blood	TEG	Negative correlation between age and K value. Positive correlation between age and α angle, MA, LY30. Positive correlation between MA and birthweight. R value of females was higher than that of males and higher in cesarean section than that of spontaneous delivery
Sokou et al. [62]	282 neonates. 198 term and 84 preterm	Whole blood	ROTEM	Enhanced fibrinolytic activity in preterm neonates (LY60, significantly lower)
Sewell et al. [61]	30 full-term neonates	Whole blood	TEG	Lower R and K values in neonates compared to older children. Higher fibrinolysis or rate of clot breakdown (LY30) and coagulation index (CI) in neonates compared to older children
Motta et al. [59]	65 preterm neonates. <32 weeks: 32 32–37 weeks: 33	Whole blood	TEG	Increased fibrinolysis (higher LY30) in early preterm neonates compared to moderate/late preterm neonates
Ravn et al. [64]	149 children. Neonates: 30 1–18 months: 72 19–72 months: 47	Whole blood	ROTEM	No sign of developmental changes in ROTEM assays, apart from CT in the EXTEM assay
Oswald et al. [97]	51 infants (0–3 months)	Whole blood	TEM	Subjects aged 0–3 months exhibited accelerated initiation and propagation of coagulation and maximum clot firmness
Kettner et al. [98]	40 neonates. 27–31 weeks: 13, 32–36 weeks: 9, 36–40 weeks: 7, 34–40 weeks corrected: 11	Whole blood	TEG	When compared with the adult group, thromboelastography revealed no defects in coagulation from groups of clinically stable infants
Miller et al. [60]	237 children Neonates: 37	Whole blood	TEG	Neonatal values did not statistically differ from those of older patients (aged from 1 to 24 months)

## Data Availability

Not applicable.
